# Decision‐making in return to sport clearance after ACL reconstruction is primarily based on objective criteria: Insights from AGA knee experts

**DOI:** 10.1002/jeo2.70662

**Published:** 2026-02-17

**Authors:** Philipp W. Winkler, Andrea E. Achtnich, Christian Schoepp, Georg Brandl, Tobias C. Drenck, Johannes Glasbrenner, Christoph Kittl, Gerd Rauch, Thomas Stein, Björn H. Drews, Lukas Willinger

**Affiliations:** ^1^ Department for Orthopaedics and Traumatology Kepler University Hospital GmbH Johannes Kepler University Linz Linz Austria; ^2^ Department of Sports Orthopedics TUM University Hospital Technical University of Munich Munich Germany; ^3^ Department of Arthroscopic Surgery Sports Traumatology and Sports Medicine, BG Klinikum Duisburg gGmbH Duisburg Germany; ^4^ Department of Orthopaedic Surgery II Herz‐Jesu Krankenhaus Vienna Austria; ^5^ Department of Trauma Surgery Orthopaedics and Sports Traumatology, BG Klinikum Hamburg Hamburg Germany; ^6^ Traumatology and Orthopaedics Mallorca ‐ TOM Palma de Mallorca Spain; ^7^ Department of Trauma, Hand and Reconstructive Surgery University Hospital Münster Münster Germany; ^8^ MVZ OCP Kassel Lichtenau gGmbH Kassel Germany; ^9^ Department of Sports Medicine and Exercise Physiology Goethe University Frankfurt Frankfurt Germany; ^10^ SPORTHOLOGICUM ‐ Knee Center Frankfurt ‐ Center for Sport and Joint Injuries Frankfurt Germany; ^11^ Department for Surgery St. Vinzenz Hospital Pfronten Pfronten Germany

**Keywords:** ACL, athlete, cruciate ligament, readiness, survey, test battery

## Abstract

**Purpose:**

To identify current surgical strategies and objective criteria used for return to sport (RTS) clearance after primary isolated anterior cruciate ligament (ACL) reconstruction, based on a survey of knee surgery experts.

**Methods:**

An online survey was developed by the ‘Knee Ligament’ committee of the German‐speaking Arthroscopy Society (AGA) and distributed to certified ‘AGA Knee Experts’ in November and December 2023. The final questionnaire consisted of 26 questions covering surgical strategies, postoperative evaluation, and RTS clearance after primary isolated ACL reconstruction. Participants were asked to rank objective RTS criteria, and weighted mean ranks were calculated to assess their relative importance (1 = most important, 8 = least important). Data were analysed descriptively to reflect current expert practices.

**Results:**

A total of 113 board‐certified knee surgeons from Germany, Austria, and Switzerland participated in this survey. Hamstring tendon autografts were the preferred graft choice for primary isolated ACL reconstruction (87%), with over half of the respondents (58%) adapting graft selection based on the patient's activity level. RTS testing is routinely performed by 64% of participants. The most frequently used RTS tests are hop tests (93%), assessment of movement quality (59%), and postural stability (56%). The most important objective criteria for RTS clearance were time since ACL reconstruction (mean rank, 2.7), manual clinical examination (mean rank, 2.7), and RTS assessment tools (mean rank, 3.2). Concomitant surgical procedures such as cartilage treatment, meniscus repair, and osteotomies affect RTS clearance. Sport psychological assessment is rarely used (12%).

**Conclusion:**

This study found that knee experts prioritised time since surgery, clinical examination, and RTS assessment tools as the most important criteria for unrestricted RTS clearance after primary isolated ACL reconstruction. RTS clearance is affected by concomitant surgical procedures, whereas psychological assessment remains uncommon.

**Level of Evidence:**

Level V.

**Abbreviations**
ACL: anterior cruciate ligament
ACL‐RSI: Anterior Cruciate Ligament – Return to Sport After Injury Scale
EQ‐5D: European Quality of Life 5 Dimensions
IKDC: International Knee Documentation Committee
KOOS: Knee Injury and Osteoarthritis Outcome Score
K‐SES: Knee Self‐Efficacy Scale
LEAP: lateral extra‐articular procedure
MRI: magnetic resonance imaging
PROMs: patient‐reported outcome measures
RTS: return to sport
TSK: Tampa Scale of Kinesiophobia

## INTRODUCTION

Young and physically active individuals are most frequently affected by anterior cruciate ligament (ACL) injuries [23]. Athletes who sustain an ACL injury often report reduced quality of life and physical performance, which may compromise their long‐term athletic career [7, 8, 29]. The management of ACL injuries has evolved rapidly over the last decades as a result of high‐quality clinical and biomechanical research [24]. ACL reconstruction has become the standard of care for young athletes with a primary ACL injury [17].

Although modern ACL reconstruction techniques result in statistically significant and clinically relevant improvements in patient‐reported outcome measures (PROMs) and return to sport (RTS) rates of 68%–82% [1, 32, 37], many athletes never return to their pre‐injury level of sports. A recent study of 1392 adolescent patients who underwent ACL reconstruction found that only 64%–76% of patients returned to their pre‐injury level of sport across a variety of sport disciplines [38]. In addition, meta‐analyses reported that only 44%–55% of patients returned to competitive sport after ACL reconstruction [1, 2]. These findings highlight the need for a more nuanced approach for return to physical activity after ACL reconstruction. Progression along the RTS continuum should be guided by specific criteria to optimise outcomes [12, 21].

Historically, the time period after ACL reconstruction has been the primary criterion for RTS. However, an international expert consensus reached 100% agreement that purely time‐based RTS decision‐making after ACL reconstruction is no longer appropriate [21]. According to the expert consensus, RTS clearance after ACL reconstruction should include objective physical examination data (100% agreement), standardised, validated and peer‐reviewed RTS tests (88% agreement), and assessment of psychological readiness (85% agreement) [21].

Although research has shown that passing an RTS test battery can reduce the risk of ACL reconstruction graft failure by 60% [35], a systematic review found that only 13% of studies used objective criteria (other than time after ACL reconstruction) for unrestricted RTS clearance [4]. Accordingly, it remains unclear whether the growing body of evidence has led to a shift from purely time‐based to criterion‐based RTS clearance after primary ACL reconstruction.

The purpose of this study was to identify current surgical strategies and objective criteria used for RTS clearance after primary isolated ACL reconstruction, based on a survey of knee surgery experts among the German‐speaking Arthroscopy Society (AGA). It was hypothesised that clearance for RTS after primary isolated ACL reconstruction would rely primarily on objective criteria such as RTS assessment tools, PROMs, and sport psychological assessments, rather than solely on time.

## METHODS

Data collection for this study was approved by the board of the AGA. The AGA is the largest European society for arthroscopy and joint surgery, comprising international members but predominantly representing Germany, Austria, and Switzerland.

An online survey was created in a multi‐stage process by the AGA committee ‘Knee Ligament’. The members of the AGA committee “Knee Ligament” are experienced orthopaedic surgeons who have a special interest in knee ligament surgery. The committee membership requires active participation in scientific and society‐related topics and is limited to three years. The first author (PWW) developed an initial survey consisting of 30 questions. After several rounds of discussion with the entire committee, four questions were removed, resulting in a final set of 26 questions that was approved by all members of the committee. The 26 questions were divided into four sections: general information, technical details, postoperative evaluation, and RTS. The survey aimed to identify specific characteristics of surgical treatment, rehabilitation, and objective criteria for RTS clearance after primary isolated ACL injury among knee surgery experts. Key questions of the survey can be found in Table 1. The entire survey can be found in Supporting Information: *German version*: Table 1 and *English Version*: Table 2 *)*. In November and December 2023, the survey was distributed by the AGA secretary to all ‘AGA Experts Knee’ using an online survey platform (SurveyMonkey Inc., San Mateo, CA, USA). Participation was voluntary, and declining to participate entailed no adverse consequences for members.

**Table 1:** Key questions from the online survey (translated into English).

Section 1: General information Q4. How many primary ACL reconstructions do you perform annually? − < 30 − 30–50 − 50–100 − 100–200 − >200
Section 2: Technical details Q6. Is your graft choice dependent on the patient's preoperative activity level? − Yes − No Q9. What do you consider the most important criteria to perform a concomitant lateral extra‐articular procedure in primary isolated ACL reconstruction? (multiple selections possible) − Patient age − Antero‐lateral rotatory knee instability (Pivot‐Shift ≥ grade 2) − Generalised ligamentous laxity (Beighton‐Score) − High preoperative activity level (Tegner Activity Scale > 6) − High posterior tibial slope (>12° according to Dejour und Bonnin) − Desire to return to high‐risk/pivoting sports − Others
Section 3: Postoperative evaluation Q15. Do you routinely perform imaging during postoperative follow‐up? − Yes − No Q19. Do you routinely collect patient‐reported outcome measures (PROMs) during postoperative follow‐up? − Yes − No Q21. Do you routinely perform return to sport tests during postoperative follow‐up? − Yes − No
Section 4: Return to sport Q24. Indicate the criteria you consider most important for assessing readiness to return to sport after isolated primary ACL reconstruction, and rank them from 1 (most important) to 8 (least important). − Time − Subjective perception of the patient − Patient‐Reported Outcome Measures − Manual clinical examination (Lachman Test, Range‐of‐Motion, etc.) − Arthrometer‐based clinical examination − Postoperative MRI − Return‐to‐Sport Assessment Tools (jumping tests, agility assessment, force measurement, etc.) − Sports psychological assessment

Abbreviations: ACL, anterior cruciate ligament; MRI, magnetic resonance imaging; PROMs, patient‐reported outcome measures; Q, question.

To obtain the 'AGA Expert Knee'” certificate, the objective criteria listed in Table 2 must be fulfilled. In addition, re‐certification is required every 5 years. Consequently, this certification is reserved for highly qualified and dedicated knee surgeons.

**Table 2:** Requirements for the ‘AGA Expert Knee’ certificate.

Surgical experience − 1000 knee arthroscopies, including 450 reconstructive or complex knee procedures (including open joint‐preserving surgical procedures)
Training − Board‐certified orthopaedic and trauma surgeon − Proof of completed standardised training, such as the AGA course curriculum for the knee
Quality assurance − Participation in the DART with registration of at least 50 patients per year or 250 patients in 5 years (Science or Basic)
Society − The certificate will only be awarded after approval by the AGA board − AGA membership of at least three years.

Abbreviations: AGA, German‐speaking Arthroscopy Society; DART, German‐speaking Arthroscopy Register.

### Statistical analysis

After the 2‐month data collection period, all responses were extracted from the online survey database and summarised. For each item, the number and percentage of respondents selecting each answer were reported. For questions allowing multiple selections, the cumulative percentages may exceed 100%. For Question 24 (Table 1), eight objective criteria for RTS clearance were provided, and participants were asked to rank them from 1 (most important criterion) to 8 (least important criterion). For each criterion, a weighted mean rank was calculated to assess its overall importance for RTS clearance. The percentage of respondents assigning a given rank (1–8) was multiplied by the respective rank number, and the corresponding products were summarised. This sum was then divided by 100 to account for the percentage scale, yielding a weighted mean rank. Lower values indicate that a criterion was more frequently ranked as important (closer to Rank 1), whereas higher values reflect less importance (closer to Rank 8).

## RESULTS

A total of 113 'AGA Experts Knee' took part in the survey, with 103 participants having successfully completed the entire survey. Most of the participants work in Germany (86%), followed by Austria (8%), and Switzerland (6%). The participants demonstrated a high level of expertise: 81% are board‐certified orthopaedic and trauma surgeons for more than 10 years, 79% perform more than 200 knee arthroscopic procedures annually, and 71% conducted over 50 ACL reconstructions per year.

**Figure 1:**
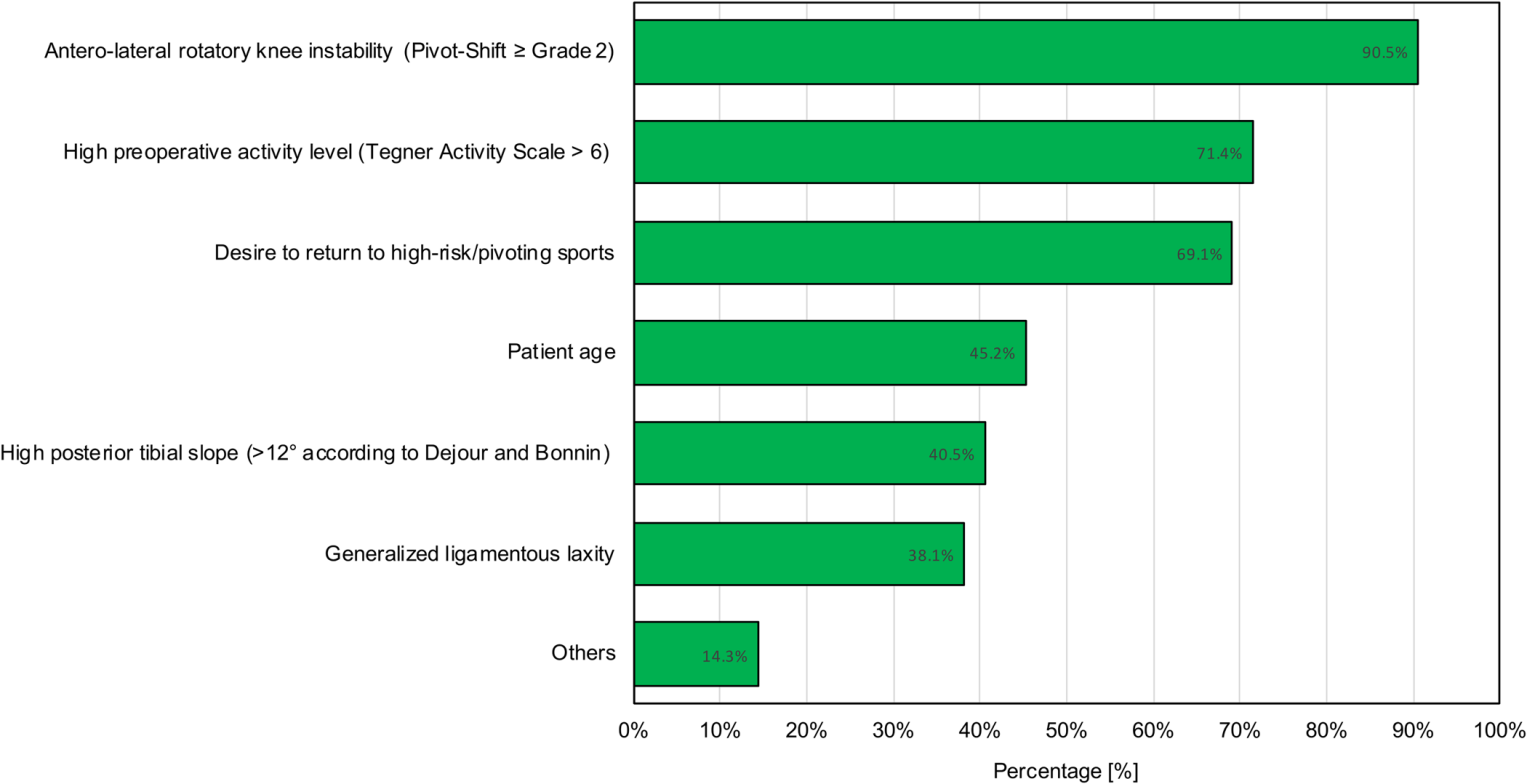
Most important criteria to perform a concomitant lateral extra‐articular procedure during primary isolated ACL reconstruction. Since more than one answer could be selected, the cumulative percentage exceeds 100%.

Primary isolated ACL reconstruction is performed using hamstring tendon autograft (87%), followed by quadriceps tendon autograft (12%), and bone‐patellar tendon‐bone autograft (2%). Fifty‐eight percent of participants select the graft based on the patient's activity level, and 38% of participants routinely perform a concomitant lateral extra‐articular procedure (LEAP) in primary isolated ACL reconstruction. On average, a concomitant LEAP is performed in 32% of primary isolated ACL reconstructions. The most important criteria to perform a concomitant LEAP are shown in Figure 1.

The following clinical tests are performed during postoperative follow‐up examination: Lachman Test (99%), Anterior Drawer Test (61%), Pivot‐Shift Test (80%), Lever‐Sign Test (5%), and others (9%). Routine imaging after primary isolated ACL reconstruction was reported by 42% of participants and most commonly included radiographs (89%), followed by magnetic resonance imaging (MRI, 22%) and other modalities (2%). Patient‐reported outcome measures are collected by 43% of participants after primary isolated ACL reconstruction and include the International Knee Documentation Committee (IKDC) Subjective Knee Form (71%), Tegner Activity Scale (66%), Knee Injury and Osteoarthritis Outcome Score (KOOS, 64%), Lysholm Score (61%), European Quality of Life 5 Dimensions (EQ‐5D, 23%), Anterior Cruciate Ligament – Return to Sport After Injury Scale (ACL‐RSI, 14%), and others (5%). Return to sport tests are performed by 64% of participants after primary isolated ACL reconstruction. The RTS test categories used are shown in Table 3.

**Table 3:** Return to sport test categories used by the participants.

Test categories	Percentage
Hop tests	92.7%
Movement quality assessment	58.8%
Postural stability assessment	55.9%
Isokinetic or isometric strength measurement	52.9%
Speed tests	45.6%
Agility tests	44.1%
Fatigue testing	19.1%
Sport psychological assessment	11.8%
Others	4.4%

*Note*: Since more than one test category could be selected, the cumulative percentage exceeds 100%. Note that the percentages refer exclusively to participants who routinely perform return to sport tests (*n* = 68).

**Figure 2:**
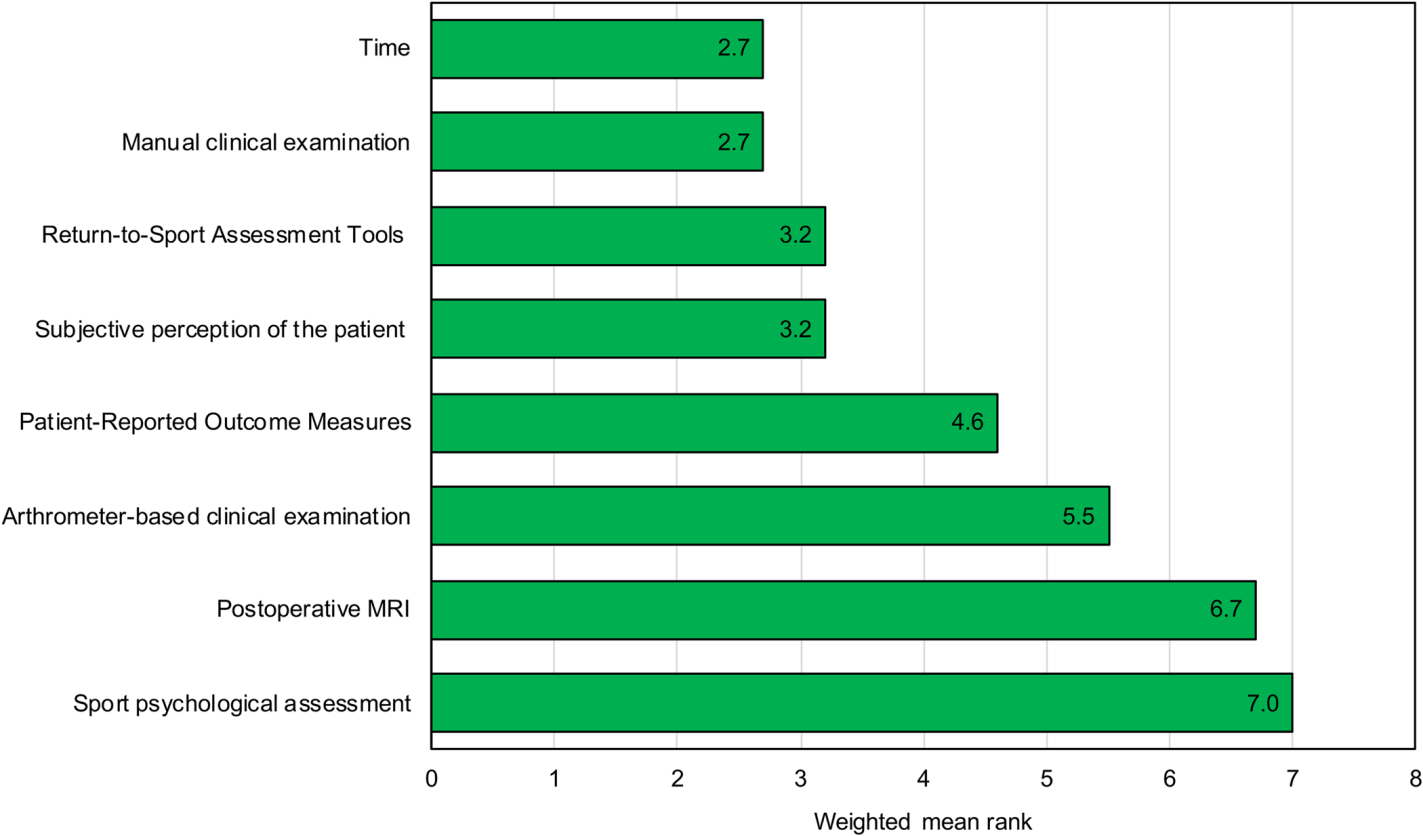
Objective criteria for return to sport clearance. Participants were asked to rank the criteria from 1 (most important criterion) to 8 (least important criterion). For each criterion, a weighted mean rank was calculated to assess its overall importance for RTS clearance. Lower values indicate that a criterion was more frequently ranked as important (closer to Rank 1), whereas higher values reflect less importance (closer to Rank 8). MRI, magnetic resonance imaging; RTS, return to sport.

Figure 2 shows the eight objective criteria used for RTS clearance along with their weighted mean ranks (note that values closer to 1 indicate greater importance). Concomitant surgical procedures such as cartilage treatment (90%), meniscus repair (85%), and osteotomies (46%–55%) affect RTS clearance after primary isolated ACL reconstruction. Sport psychological assessment is performed by 12% of participants prior to RTS clearance after primary isolated ACL reconstruction.

All results of the survey are presented in detail in Supporting Information: *German version*: Table 1 and *English Version*: Table 2.

## DISCUSSION

The most important finding of this study was that highly experienced knee surgeons prioritised time since ACL reconstruction, manual clinical examination (i.e., Lachman Test, Range‐of‐Motion, etc.), and RTS assessment tools as the most important criteria to determine readiness to unrestricted RTS after primary isolated ACL reconstruction. More subjective measures such as PROMs and patients' own perception of RTS readiness, were considered secondary factors. Instrumented laxity testing (i.e., arthrometer‐based clinical examination), postoperative MRI, and sport psychological assessment were generally ranked lower. This ranking likely reflects their more limited routine availability and utilisation in daily clinical practice rather than a lack of clinical relevance. Overall, the main findings of the survey contradict the primary hypothesis of the study.

Modern ACL reconstruction techniques have been associated with favourable clinical outcomes and high levels of patient satisfaction [25, 37]. While RTS rates of 68%–82% have been reported [1, 32], a more detailed analysis of the RTS continuum reveals that only 63%–76% return to their pre‐injury level of sport, and only 44%–55% return to competitive sport [1, 2, 38]. Premature or undefined RTS clearance may contribute to these suboptimal outcomes by increasing the risk of early ACL reconstruction graft failure. A systematic review demonstrated that passing specific RTS criteria after ACL reconstruction can reduce the risk of ACL graft failure by 60% [35]. The importance of objective criteria for RTS clearance after ACL reconstruction was further underscored by an expert consensus, in which 100% of participants agreed that purely time‐based decision‐making is no longer appropriate and that more objective measures should be incorporated into the RTS clearance process [21]. Unfortunately, systematic reviews indicate that 22%–32% of studies relied purely on time since ACL reconstruction to determine RTS clearance [4, 28]. However, objective criteria were applied in 63% of studies, and 52% used more than one criterion for RTS clearance [28].

In this study, knee experts from Germany, Austria, and Switzerland identified time since ACL reconstruction, manual clinical examination, and RTS assessment tools as the most important criteria for RTS clearance after primary isolated ACL reconstruction. In contrast, subjective measures such as patient perception, PROMs, and psychological assessment as well as objective measures like instrumented laxity testing and postoperative MRI were considered less important. Although time since ACL reconstruction was considered as one of the most important criteria in this survey, it should be noted that less than 50% of participants conducted follow‐ups beyond 6 months after ACL reconstruction. The ACL graft undergoes a complex biological maturation process, commonly referred to as ligamentization, which substantially affects its biomechanical properties. Experimental and clinical data suggest that this process may take up to 12–24 months for the graft to approach the strength of the native ACL, during which the graft remains particularly vulnerable to re‐rupture [6, 19]. A clinical study has demonstrated that the risk of ACL graft failure decreases by more than 50% for each month RTS is delayed up to nine months after ACL reconstruction [13]. Despite this protective effect, time since surgery alone is increasingly recognised as a poor and non‐objective indicator of successful and safe RTS [21, 34]. Accordingly, recent systematic review advocate for a multifactorial RTS decision‐making approach that combines time‐based criteria with objective, criteria‐based functional and strength assessments. Incorporating at least two functional performance tests in addition to strength testing and a minimum time threshold has been shown to reduce ACL graft failure rates from 2%–11% to 2%–8%, while simultaneously increasing RTS rates from 52%–90% to 73%–90% [33]. Taken together, time since surgery should be regarded as an important factor, particularly a minimum RTS timeframe, rather than a standalone RTS criterion. Instead, RTS decisions should be based on a comprehensive, multifactorial framework integrating time, functional and strength testing, and patient‐specific factors such as age and sex. However, it is encouraging that nearly two‐thirds (64%) of experts routinely perform RTS testing after primary isolated ACL reconstruction. This suggests that the growing body of evidence supporting RTS testing is increasingly being translated into clinical practice.

Return to sport assessment tools evaluate aim to evaluate patients' functional performance and provide objective data to support RTS clearance decisions. Multidimensional test batteries have been developed to assess muscle strength, agility, movement quality, speed, and coordination [11, 16, 20]. Jump‐based assessments represent key components of such RTS test batteries, with the counter movement jump and the drop jump test being the most commonly applied tests [15, 16]. Although research has demonstrated the value of jump tests as part of RTS decision‐making [13, 14], the optimal timing of RTS testing after ACL reconstruction remains unclear [11, 18, 20]. Importantly, early postoperative testing, particularly within the first three months after ACL reconstruction, should not be an indicator for unrestricted RTS. Rather, such assessments may provide information on functional recovery and rehabilitation progress, but cannot be used in isolation to determine RTS readiness at this stage. In a prospective study, only 16% and 17% of patients fulfilled predefined objective RTS criteria at a mean of 171 and 239 days after ACL reconstruction, respectively. The most limiting factor was insufficient limb symmetry (<90% for the dominant leg and <80% for the non‐dominant leg) during jumping tests [15]. These findings highlight that neither RTS test results alone nor a specific postoperative time point guarantees a safe RTS. Instead, RTS clearance should be based on a comprehensive, multifactorial evaluation incorporating time since surgery, functional performance, clinical examination, and individual patient‐specific factors.

Sport psychological assessment, including the evaluation of psychological readiness, kinesiophobia, and fear of re‐injury, has gained increasing attention in the RTS clearance after ACL reconstruction. Commonly used instruments include the ACL Return to Sport after Injury Scale (ACL‐RSI) [36], the Tampa Scale of Kinesiophobia (TSK) [9], or the Knee Self‐Efficacy Scale (K‐SES) [31]. A recent systematic review demonstrated a negative correlation between the degree of kinesiophobia and PROMs after ACL reconstruction [22]. In addition, successful RTS after ACL reconstruction was found the be associated with better psychological metrics meaning a higher degree of self‐efficacy and psychological readiness and a lower level of kinesiophobia [39]. Despite this evidence, sports psychological assessment was ranked as the least important criterion for RTS clearance in the present study and was performed by only 12% of participants. This discrepancy may be explained by limited routine availability, time constraints, and the need for specialised expertise. In addition, conflicting evidence regarding the direct impact of psychological assessments on RTS outcomes may further limit their widespread implementation in daily clinical practice [27].

Patients with ACL injuries frequently present with concomitant meniscal, chondral, or ligamentous injuries, many of which require surgical management. A recent expert consensus emphasised that such injuries should be considered in the RTS decision‐making process after ACL reconstruction (96% agreement) [21]. According to this survey, participants confirmed that additional surgical procedures performed at the time of ACL reconstruction, especially cartilage repair, meniscus repair, and osteotomies, should be considered for RTS clearance.

Eighty‐seven percent of participants in this survey reported using hamstring tendon autograft as the graft of choice for primary isolated ACL reconstruction. Additionally, 58% indicated that graft choice depends on the patient's activity level. In addition, 38% of experts reported performing LEAP in an average of 32% of patients undergoing primary isolated ACL reconstruction. The most important criteria to perform combined LEAP and ACL reconstruction are antero‐lateral rotatory knee instability (pivot‐shift ≥ grade 2), followed by high preoperative activity level (Tegner Activity Scale > 6), the desire to return to high‐risk/pivoting sports, patient age, a posterior tibial slope > 12°, and generalised ligamentous laxity (Figure 1). These criteria align with a recently published international consensus statement on the indications for LEAP in ACL reconstruction [30]. The relevance of these criteria and the use of LEAP in primary ACL reconstruction was highlighted in a randomised controlled trial comparing hamstring tendon ACL reconstruction with (*n* = 291) and without (*n* = 298) LEAP. The authors found that patients who underwent combined ACL reconstruction and LEAP had a significantly lower risk of ACL graft failure than patients who underwent isolated ACL reconstruction (4% vs. 11%) [10]. These findings were confirmed by other studies [3, 5], which could also show a higher RTS rate after combined ACL reconstruction and LEAP [26].

This study offers valuable insights into the management and RTS clearance following primary isolated ACL reconstruction. However, several limitations must be acknowledged. As a survey‐based study, the results reflect an expert opinion rather than a formal consensus. Still, the inclusion of over 100 knee experts from various countries and clinical settings enhances the relevance and applicability of the findings to daily clinical practice. Another limitation is that selection bias needs to be considered when interpreting the results of this survey. Surgeons who participated in this survey might differ in their clinical approach or interest in the topic compared to non‐responders. Furthermore, it should be acknowledged that manual clinical examination is non‐instrumented and examiner‐dependent, and therefore does not constitute a fully objective measurement in the methodological sense.

## CONCLUSIONS

This study found that knee experts prioritised time since surgery, clinical examination, and RTS assessment tools as the most important criteria for unrestricted RTS clearance after primary isolated ACL reconstruction. RTS clearance is affected by concomitant surgical procedures, whereas psychological assessment remains uncommon. This suggests that, despite increasing evidence supporting more comprehensive RTS assessment tools, there is still a need for clear, standardised, and clinically applicable RTS criteria.

## AUTHOR CONTRIBUTIONS

All listed authors have contributed substantially to this work: Christian Schoepp, Georg Brandl, Tobias C. Drenck, Johannes Glasbrenner, Christoph Kittl, Gerd Rauch, Thomas Stein, Björn H. Drews, Lukas Willinger and Philipp W. Winkler developed the survey. Philipp W. Winkler collected data, performed statistical analysis, literature review, and primary manuscript preparation. Christian Schoepp, Georg Brandl, Tobias C. Drenck, Johannes Glasbrenner, Christoph Kittl, Gerd Rauch, Thomas Stein, Björn H. Drews, and Lukas Willinger assisted with interpretation of the results as well as editing and final manuscript preparation. All authors read and approved the final manuscript.

## CONFLICTS OF INTEREST STATEMENT

Philipp W. Winkler works as Web Editor for Knee Surgery Sports Traumatology Arthroscopy (KSSTA). Björn H. Drews is consultant to Arthrex Inc. and Medacta. Andrea Achtnich is consultant to Arthrex Inc. and receives honoraria from Arthrex Inc., Ao foundation and Medi GmbH.

## ETHICS STATEMENT

The authors have nothing to report.

## Supporting information

Supplement 1 clean.

## Data Availability

Data are available from the corresponding author upon reasonable request.
