# Supplementary material for: Decision‐making in return to sport clearance after ACL reconstruction is primarily based on objective criteria: Insights from AGA knee experts
Source: J Exp Orthop. 2026 Feb 17;13(1):e70662. doi: 10.1002/jeo2.70662 (PMC12910401; doi:10.1002/jeo2.70662)
Supplement: Supplementary file 1 — Supplement 1 clean. [file JEO2-13-e70662-s001.docx]

**Supplement Table 1: Original survey in German, including responses from the members of the German-speaking Arthroscopy Society AGA^a^**

| **Block 1: Allgemein / Erfahrung**  **Q1. In welchem Land sind Sie beruflich tätig?** (n = 113)   1. Deutschland (**85.8%**) 2. Österreich (**8.0%**) 3. Schweiz (**6.2%**) 4. Anderes (**0%**) |
| --- |
| **Q2. Seit wie vielen Jahren sind Sie Facharzt?** (n = 112)   1. < 5 (**2.7%**) 2. 5-10 (**16.1%**) 3. 10-20 (**40.2%**) 4. > 20 (**41%**) 5. Ich bin nicht Facharzt (**0%**) |
| **Q3. Wie viele arthroskopische Eingriffe führen Sie jährlich durch?** (n = 113)   1. < 50 (**0%**) 2. 50-100 (**5.3%**) 3. 100-200 (**15.9%**) 4. 200-500 (**45.1%**) 5. > 500 (**33.6%**) |
| **Q4. Wie viele primäre VKB-Rekonstruktionen führen Sie jährlich durch?** (n = 113)   1. < 30 (**6.2%**) 2. 30-50 (**23.0%**) 3. 50-100 (**24.8%**) 4. 100-200 (**23.9%**) 5. > 200 (**22.1%**) |
| **Block 2: Technische Details**  **Q5. Welches Transplantat bevorzugen Sie zur isolierten primären VKB-Rekonstruktion?** (n = 111)   1. Hamstringsehnen (**86.5%**) 2. Quadrizepssehne (mit o. ohne Knochenblock) (**11.7%**) 3. Patellarsehne (mit o. ohne Knochenblock) (**1.8%**) 4. Peroneussehne (**0%**) 5. Rectus femoris Sehne (**0%**) 6. Allograft (**0%**) 7. Anderes (**0%**) |
| **Q6. Ist Ihre Transplantatwahl abhängig vom präoperativen Aktivitätsniveau der Patientin / des Patienten?** (n = 111)   1. Ja (**57.7%**) 2. Nein (**42.3%**) |
| **Q7. Führen Sie regelmäßig eine additive anterolaterale Stabilisierung im Rahmen einer primären VKB-Rekonstruktion durch?** (n = 111)   1. Ja (**37.8%**) 2. Nein (**62.2%**)   Falls ja: |
| **Q8. Bei wieviel Prozent Ihrer Patient:innen führen Sie eine additive anterolaterale Stabilisierung im Rahmen einer primären VKB-Rekonstruktion durch?** (n = 42)   1. **32%** (Durchschnitt) |
| **Q9^b^. Was sind für Sie die wichtigsten Kriterien zur Indikationsstellung einer additiven anterolateralen Stabilisierung im Rahmen einer primären VKB-Rekonstruktion? (Mehrfachauswahl möglich)** (n = 42)   1. Patient:innenalter (**45.2%**) 2. Anterolaterale Rotationsinstabilität (Pivot-Shift ≥ Grad 2) (**90.5%**) 3. Generelle Hyperlaxizität (Beighton-Score) (**38.1%**) 4. Hohes präoperatives Aktivitätsniveau (Tegner Aktivitätsskala > 6) (**71.4%**) 5. Hoher tibialer Slope (>12 Grad entsprechend der Mesmethode nach Dejour und Bonnin) (**40.5%**) 6. Wunsch zur Rückkehr zu Hochrisiko-Sportarten bzw. Pivoting-Sportarten (**69.1%**) 7. Weitere (**14.3%**) |
| **Q10. Empfehlen Sie Ihren Patient:innen eine Prähabilitation vor einer primären VKB-Rekonstruktion?** (n = 111)   1. Ja (**68.5%**) 2. Nein (**31.5%**) |
| **Q11. Empfehlen Sie das Tragen einer Kniegelenksorthese nach einer isolierten primären VKB-Rekonstruktion?** (n = 111)   1. Ja (**75.7%**) 2. Nein (**24.3%**)   Falls ja: |
| **Q12. Über welchen Zeitraum nach einer isolierten primären VKB-Rekonstruktion empfehlen Sie das Tragen einer Kniegelenksorthese?** (n = 84)   1. 2 Wochen postoperativ (**3.6%**) 2. 6 Wochen postoperativ (**76.2%**) 3. 3 Monate postoperativ (**11.9%**) 4. 6 Monate postoperativ (**4.8%**) 5. Anders (**3.6%**) |
| **Block 3: Postoperative Evaluation**  **Q13^b^. Wann führen Sie routinemäßige Verlaufskontrollen nach einer isolierten primären VKB-Rekonstruktion durch? (Mehrfachauswahl möglich)** (n = 109)   1. 6 Wochen postoperativ (**89.9%**) 2. 3 Monate postoperativ (**68.8%**) 3. 6 Monate postoperativ (**69.7%**) 4. 9 Monate postoperativ (**22.9%**) 5. 12 Monate postoperativ (**37.6%**) 6. 24 Monate postoperativ (**4.6%**) 7. Weitere (**10.1%**) |
| **Q14^b^. Welche klinischen Tests zur Überprüfung des VKB führen Sie routinemäßig im postoperativen Follow-up nach einer isolierten primären VKB-Rekonstruktion durch? (Mehrfachauswahl möglich)** (n = 109)   1. Lachman Test (**99.1%**) 2. Schubladen Test (**60.6%**) 3. Pivot-Shift Test (**79.8%**) 4. Lever-Sign Test (**4.6%**) 5. Weitere (**9.2%**) |
| **Q15. Führen Sie eine routinemäßige Bildgebung im postoperativen Follow-up durch?** (n = 109)   1. Ja (**42.2%**) 2. Nein (**57.8%**) |
| **Q16^b^. Welche Bildgebung(en) führen Sie routinemäßig im postoperativen Follow-up durch? (Mehrfachauswahl möglich)** (n = 46)   1. Röntgen (**89.1%**) 2. MRT (**21.7%**) 3. CT (**0%**) 4. Andere (**2.2%**) |
| **Q17. Führen Sie routinemäßige Untersuchungen mit einem Arthrometer (instrumentelle Stabilitätstestung) im postoperativen Follow-up durch?** (n = 109)   1. Ja (**32.1%**) 2. Nein (**67.9%**) |
| Falls ja:  **Q18^b^. Welche Arthrometer verwenden Sie routinemäßig im postoperativen Follow-up? (Mehrfachauswahl möglich)?** (n = 35)   1. KT-1000/2000 (**54.3%**) 2. Rolimeter (**57.1%**) 3. KLT (Knielaxizitätstester) (**8.6%**) 4. KiRa (Kinematic Rapid Assessment) (**0%**) 5. PIVOT iPad Application (**0%**) 6. Anderes (**5.7%**) |
| **Q19. Erheben Sie routinemäßig Patient-Reported Outcome Measures (PROMs) im postoperativen Follow-up?** (n = 109)   1. Ja (**43.1%**) 2. Nein (**56.9%**)   Falls ja:  **Q20^b^. Welche Patient-Reported Outcome Measures (PROMs) erheben Sie routinemäßig im postoperativen Follow-up? (Mehrfachauswahl möglich)** (n = 44)   1. Lysholm Score (**61.4%**) 2. International Knee Documentation Committee (IKDC) Subjective Knee Form (**70.5%**) 3. Knee Injury and Osteoarthritis Outcome Score (KOOS) (**63.6%**) 4. Tegner Aktivitätsskala (**65.9%**) 5. Anterior Cruciate Ligament – Return to Sport After Injury Scale (ACL-RSI) (**13.6%**) 6. European Quality of Life 5 Dimensions (EQ-5D) (**22.7%**) 7. Andere (**4.6%**)   **Q21. Führen Sie routinemäßige Return-to-Sport Testungen im postoperativen Follow-up durch?** (n = 107)   1. Ja (**63.6%**) 2. Nein (**36.5%**)   Falls ja:  **Q22^b^. Welche Return-to-Sport Testungen führen Sie routinemäßig im postoperativen Follow-up durch? (Mehrfachauswahl möglich)** (n = 68)   1. Sprungtests (z.B.: Drop Jump, Counter Movement Jump, Side Hop Test, etc.) (**92.7%**) 2. Tests zur posturalen Kontrolle (z.B.: Star Excursion Balance Test, etc.) (**55.9%**) 3. Schnelligkeitstests (z.B.: Tapping Test, etc.) (**45.6%**) 4. Agilitätstests (z.B.: Agility T-Test, Tests am Speedcourt, etc.) (**44.1%**) 5. Tests zur Provokation der Ermüdung (**19.1%**) 6. Tests zur Beurteilung der Bewegungsqualität (z.B.: Kontrolle der Beinachse während Drop Jump, etc.) (**58.8%**) 7. Isokinetische o. isometrische Kraftmessung (**52.9%**) 8. Sportpsychologische Untersuchung (z.B.: ACL-RSI) (**11.8%**) 9. Andere (**4.4%**)   **Q23. Verwenden Sie routinemäßig digitale Gesundheitsanwendungen (DiGAs) im postoperativen Follow-up nach isolierter primärer VKB-Rekonstruktion?** (n = 107)   1. Ja (**63.6%**) 2. Nein (**36.5%**)   **Block 4: Return-to-Sport**  **Q24. Nennen Sie die für Sie wichtigsten Kriterien zur Beurteilung der Sportfähigkeit nach isolierter primärer VKB-Rekonstruktion? (Erstellen Sie eine Reihung: 1 = wichtigstes Kriterium, 8 = unwichtigstes Kriterium)?** (n = 103)   1. Zeit 2. Subjektives Befinden der Patient:innen 3. Patient-Reported Outcome Measures 4. Manuelle klinische Untersuchung (Lachman Test, Range-of-Motion, etc.) 5. Arthrometer-gestützte klinische Untersuchung 6. Postoperatives MRT 7. Return-to-Sport Assessment Tools (Sprungtests, Agilitätstests, Kraftmessung, etc.) 8. Sportpsychologische Untersuchung  \| **Rang** \| **1** \| **2** \| **3** \| **4** \| **5** \| **6** \| **7** \| **8** \| \| --- \| --- \| --- \| --- \| --- \| --- \| --- \| --- \| --- \| \| **Zeit** \| 44.7% \| 10.7% \| 13.6% \| 10.7% \| 11.7% \| 5.8% \| 1.0% \| 1.9% \| \| **Subjektives Befinden der Patient:innen** \| 7.8% \| 26.2% \| 27.2% \| 19.4% \| 14.6% \| 1.9% \| 2.9% \| 0% \| \| **PROMs** \| 0% \| 9.7% \| 8.7% \| 29.1% \| 28.2% \| 16.5% \| 5.8% \| 1.9% \| \| **Klinische Untersuchung** \| 19.4% \| 29.1% \| 27.2% \| 13.6% \| 8.7% \| 1.9% \| 0% \| 0% \| \| **Arthrometer** \| 0% \| 5.8% \| 5.8% \| 5.8% \| 17.5% \| 42.7% \| 20.4% \| 1.9% \| \| **MRT** \| 0% \| 1.0% \| 1.0% \| 1.0% \| 3.9% \| 10.7% \| 42.7% \| 39.8% \| \| **RTS Assessment Tools** \| 28.2% \| 15.5% \| 14.6% \| 13.6% \| 11.7% \| 9.7% \| 6.8% \| 0% \| \| **Sportpsychologische Untersuchung** \| 0% \| 1.9% \| 1.9% \| 6.8% \| 3.9% \| 10.7% \| 20.4% \| 54.4% \|   **Q25^b^. Welche der folgenden Zusatzeingriffe im Rahmen einer primären VKB-Rekonstruktion beeinflusst für Sie die Freigabe zu Hochrisiko-Sportarten bzw. Pivoting-Sportarten? (Mehrfachauswahl möglich)** (n = 103)   1. Meniskusteilresektion (**10.7%**) 2. Meniskusnaht (**84.5%**) 3. Knorpelregeneratives bzw. -ersetzendes Verfahren (**90.3%**) 4. Anterolaterale Stabilisierung (**25.2%**) 5. Anteromediale Stabilisierung (**21.4%**) 6. Koronare Umstellungsosteotomie (z.B.: medial öffnende HTO) (**55.3%**) 7. Sagittale Umstellungsosteotomie (z.B.: Slope reduzierende Osteotomie) (**45.6%**)   **Q26. Führen Sie routinemäßige sportpsychologische Untersuchungen (Analyse von Motivation, Emotion, Wahrnehmung, Konzentration, Entscheidungsfähigkeit, sozialer Interaktion, Stress etc. im Sport) vor der Freigabe zur Sportrückkehr nach isolierter primärer VKB-Rekonstruktion durch?** (n = 103)   1. Ja (**11.7%**) 2. Nein (**88.4%**) |

^a^ ”n” indicates the number of respondents per question. The percentages in parentheses indicate the frequency of the corresponding response among the respondents; ^b^Since more than one answer could be selected, the cumulative percentage may exceed 100%.

**Supplement Table 2: Survey translated into English, including responses from the members of the German-speaking Arthroscopy Society AGA^a^**

| **Block 1: General Information / Experience**  **Q1. In which country are you professionally active?** (n = 113)   1. Germany (**85.8%**) 2. Austria (**8.0%**) 3. Switzerland (**6.2%**) 4. Other (**0%**) |
| --- |
| **Q2. For how many years have you been a board-certified specialist?** (n = 112)   1. < 5 (**2.7%**) 2. 5-10 (**16.1%**) 3. 10-20 (**40.2%**) 4. > 20 (**41%**) 5. I am not board-certified (**0%**) |
| **Q3. How many arthroscopic procedures do you perform per year?** (n = 113)   1. < 50 (**0%**) 2. 50-100 (**5.3%**) 3. 100-200 (**15.9%**) 4. 200-500 (**45.1%**) 5. > 500 (**33.6%**) |
| **Q4. How many primary ACL reconstructions do you perform per year?** (n = 113)   1. < 30 (**6.2%**) 2. 30-50 (**23.0%**) 3. 50-100 (**24.8%**) 4. 100-200 (**23.9%**) 5. > 200 (**22.1%**) |
| **Block 2: Technical Details**  **Q5. Which graft do you prefer for isolated primary ACL reconstruction?** (n = 111)   1. Hamstring tendons (**86.5%**) 2. Quadriceps tendon (with and without bone block) (**11.7%**) 3. Patellar tendon (with and without bone block) (**1.8%**) 4. Peroneus tendon (**0%**) 5. Rectus femoris tendon (**0%**) 6. Allograft (**0%**) 7. Other (**0%**) |
| **Q6. Is your graft choice dependent on the patient’s preoperative activity level?** (n = 111)   1. Yes (**57.7%**) 2. No (**42.3%**) |
| **Q7. Do you routinely perform an additional lateral extra-articular procedure during primary ACL reconstruction?** (n = 111)   1. Yes (**37.8%**) 2. No (**62.2%**)   If yes: |
| **Q8. In what percentage of your patients do you perform an additional lateral extra-articular procedure during primary ACL reconstruction?** (n = 42)   1. **32%** (Average) |
| **Q9^b^. What are the most important criteria for an additional lateral extra-articular procedure during primary ACL reconstruction? (Multiple answers possible)** (n = 42)   1. Patient age (**45.2%**) 2. Anterolateral rotatory knee instability (Pivot-Shift ≥ Grade 2) (**90.5%**) 3. Generalized ligamentous laxity (Beighton-Score) (**38.1%**) 4. High preoperative activity level (Tegner Activity Scale > 6) (**71.4%**) 5. High posterior tibial slope (>12 degress according to Dejour and Bonnin) (**40.5%**) 6. Desire to returnt to high-risk or pivoting sports (**69.1%**) 7. Other (**14.3%**) |
| **Q10. Do you recommend prehabilitation prior to primary ACL reconstruction?** (n = 111)   1. Yes (**68.5%**) 2. No (**31.5%**) |
| **Q11. Do you recommend wearing a knee brace after primary isolated ACL reconstruction?** (n = 111)   1. Yes (**75.7%**) 2. No (**24.3%**)   If yes: |
| **Q12. For how long after primary isolated ACL reconstruction do you recommend wearing a knee brace?** (n = 84)   1. 2 weeks postoperatively (**3.6%**) 2. 6 weeks postoperatively (**76.2%**) 3. 3 months postoperatively (**11.9%**) 4. 6 months postoperatively (**4.8%**) 5. Other (**3.6%**) |
| **Block 3: Postoperative Assessment**  **Q13^b^. When do you perform follow-up assessments after primary isolated ACL reconstruction? (Multiple answers possible)** (n = 109)   1. 6 weeks postoperatively (**89.9%**) 2. 3 months postoperatively (**68.8%**) 3. 6 months postoperatively (**69.7%**) 4. 9 months postoperatively (**22.9%**) 5. 12 months postoperatively (**37.6%**) 6. 24 months postoperatively (**4.6%**) 7. Other (**10.1%**) |
| **Q14^b^. Which clinical tests do you perform during follow-up assessment after primary isolated ACL reconstruction? (Multiple answers possible)** (n = 109)   1. Lachman test (**99.1%**) 2. Anterior drawer test (**60.6%**) 3. Pivot-Shift test (**79.8%**) 4. Lever-Sign test (**4.6%**) 5. Other (**9.2%**) |
| **Q15. Do you perform routine imaging during postoperative follow-up?** (n = 109)   1. Yes (**42.2%**) 2. No (**57.8%**) |
| **Q16^b^. Which imaging modalities do you use during postoperative follow-up? (Multiple answers possible)** (n = 46)   1. Radiographs (**89.1%**) 2. MRI (**21.7%**) 3. CT (**0%**) 4. Other (**2.2%**) |
| **Q17. Do you perform instrumented laxity testing during postoperative follow-up?** (n = 109)   1. Yes (**32.1%**) 2. No (**67.9%**) |
| If yes:  **Q18^b^. Which device do you use for instrumented laxity testing during postoperative follow-up? (Multiple answers possible)?** (n = 35)   1. KT-1000/2000 (**54.3%**) 2. Rolimeter (**57.1%**) 3. KLT (Knielaxizitätstester) (**8.6%**) 4. KiRa (Kinematic Rapid Assessment) (**0%**) 5. PIVOT iPad Application (**0%**) 6. Other (**5.7%**) |
| **Q19. Do you collect Patient-Reported Outcome Measures (PROMs) during postoperative follow-up?** (n = 109)   1. Yes (**43.1%**) 2. No (**56.9%**)   If yes:  **Q20^b^. Which Patient-Reported Outcome Measures (PROMs) do you collect during postoperative follow-up? (Multiple answers possible)** (n = 44)   1. Lysholm Score (**61.4%**) 2. International Knee Documentation Committee (IKDC) Subjective Knee Form (**70.5%**) 3. Knee Injury and Osteoarthritis Outcome Score (KOOS) (**63.6%**) 4. Tegner Activity Scale (**65.9%**) 5. Anterior Cruciate Ligament – Return to Sport After Injury Scale (ACL-RSI) (**13.6%**) 6. European Quality of Life 5 Dimensions (EQ-5D) (**22.7%**) 7. Other (**4.6%**)   **Q21. Do you routinely perform return-to-sport assessment during postoperative follow-up?** (n = 107)   1. Yes (**63.6%**) 2. No (**36.5%**)   If yes:  **Q22^b^. Which return-to-sport assessment tools do you perform during postoperative follow-up? (Multiple answers possible)** (n = 68)   1. Hop tests (e.g., Drop Jump, Counter Movement Jump, Side Hop Test, etc.) (**92.7%**) 2. Postural control tests (e.g., Star Excursion Balance Test, etc.) (**55.9%**) 3. Speed tests (e.g., Tapping Test, etc.) (**45.6%**) 4. Agility tests (e.g., Agility T-test, etc.) (**44.1%**) 5. Fatigue tests (**19.1%**) 6. Tests to assess the movement quality (e.g., evaluation of lower limb alignment during dromp jump, etc.) (**58.8%**) 7. Isokinetic or isometric strength testing (**52.9%**) 8. Sports psychological assessment (e.g., ACL-RSI) (**11.8%**) 9. Other (**4.4%**)   **Q23. Do you use digital health applications (DiGAs) during postoperative follow-up after isolated primary ACL reconstruction?** (n = 107)   1. Yes (**63.6%**) 2. No (**36.5%**)   **Block 4: Return-to-Sport**  **Q24. Which criteria do you consider most important for the assessment of readiness to return-to-sport after isolated primary ACL reconstruction? (Please rank the criteria: 1 = most important, 8 = least important)?** (n = 103)   1. Time since ACL reconstruction 2. Patients’ subjective perception 3. Patient-Reported Outcome Measures 4. Manual clinical examination (Lachman test, Range-of-Motion, etc.) 5. Instrumented laxity testing 6. Postoperative MRI 7. Return-to-sport assessment tools (Hop tests, agility tests, strength measurement, etc.) 8. Sports psychological assessment  \| **Rang** \| **1** \| **2** \| **3** \| **4** \| **5** \| **6** \| **7** \| **8** \| \| --- \| --- \| --- \| --- \| --- \| --- \| --- \| --- \| --- \| \| **Time since ACL reconstruction** \| 44.7% \| 10.7% \| 13.6% \| 10.7% \| 11.7% \| 5.8% \| 1.0% \| 1.9% \| \| **Patients’subjective perception** \| 7.8% \| 26.2% \| 27.2% \| 19.4% \| 14.6% \| 1.9% \| 2.9% \| 0% \| \| **PROMs** \| 0% \| 9.7% \| 8.7% \| 29.1% \| 28.2% \| 16.5% \| 5.8% \| 1.9% \| \| **Manual clinical examination** \| 19.4% \| 29.1% \| 27.2% \| 13.6% \| 8.7% \| 1.9% \| 0% \| 0% \| \| **Instrumented laxity testing** \| 0% \| 5.8% \| 5.8% \| 5.8% \| 17.5% \| 42.7% \| 20.4% \| 1.9% \| \| **MRI** \| 0% \| 1.0% \| 1.0% \| 1.0% \| 3.9% \| 10.7% \| 42.7% \| 39.8% \| \| **RTS assessment tools** \| 28.2% \| 15.5% \| 14.6% \| 13.6% \| 11.7% \| 9.7% \| 6.8% \| 0% \| \| **Sports psychological assessment** \| 0% \| 1.9% \| 1.9% \| 6.8% \| 3.9% \| 10.7% \| 20.4% \| 54.4% \|   **Q25^b^. Which of the following concomitant surgical procedures during primary ACL reconstruction affect clearance for return to high-risk or pivoting sports? (Multiple answers possible)** (n = 103)   1. Partial meniscus resection (**10.7%**) 2. Meniscus repair (**84.5%**) 3. Cartilage surgery (**90.3%**) 4. Lateral extra-articular procedure (**25.2%**) 5. Anteromedial extra-articular procedure (**21.4%**) 6. Varus/valgus osteotomy (e.g., medial opening wedge HTO) (**55.3%**) 7. Sagittal osteotomy (e.g., Tibial slope reducing osteotomy) (**45.6%**)   **Q26. Do you routinely perform sports psychological assessments (analysis of motivation, emotions, perception, concentration, decision-making, social interaction, stress, etc. in sports) before return-to-sport clearance after isolated primary ACL reconstruction?** (n = 103)   1. Yes (**11.7%**) 2. No (**88.4%**) |

^a^ ”n” indicates the number of respondents per question. The percentages in parentheses indicate the frequency of the corresponding response among the respondents; ^b^Since more than one answer could be selected, the cumulative percentage may exceed 100%.
